# Predictability reduces event file retrieval

**DOI:** 10.3758/s13414-022-02637-6

**Published:** 2022-12-28

**Authors:** Philip Schmalbrock, Bernhard Hommel, Alexander Münchau, Christian Beste, Christian Frings

**Affiliations:** 1 Department of Psychology, Cognitive Psychology, University of Trier, Universitätsring 15, DE-54296 Trier, Germany; 2 Faculty of Medicine, University Neuropsychology Center, TU Dresden, Dresden, Germany; 3 Institute of Systems Motor Science, University of Lübeck, Lübeck, Germany; 4 Department of Child and Adolescent Psychiatry, Cognitive Neurophysiology, Faculty of Medicine, TU Dresden, Dresden, Germany

**Keywords:** S–R binding, Predictability, Curiosity

## Abstract

There is growing consensus that stimulus–response bindings (event files) play a central role in human action control. Here, we investigated how the integration and the retrieval of event files are affected by the predictability of stimulus components of event files. We used the distractor–response binding paradigm, in which nominally task-irrelevant distractors are repeated or alternated from a prime to a probe display. The typical outcome of these kinds of tasks is that the effects of distractor repetition and response repetition interact: Performance is worse if the distractor repeats but the response does not, or vice versa. This partial-repetition effect was reduced when the distractor was highly predictable (Experiment 1). Separate manipulations of distractor predictability in the prime and probe trial revealed that this pattern was only replicated if the probe distractors were predictable (Experiment 2b, 3), but not if prime distractors were predictable (Experiment 2a). This suggests that stimulus predictability does not affect the integration of distractor information into event files, but the retrieval of these files when one or more of the integrated features are repeated. We take our findings to support theoretical claims that integration and retrieval of event files might differ concerning their sensitivity to top-down factors.

**Supplementary Information:**

The online version contains supplementary material available at 10.3758/s13414-022-02637-6.

## Introduction

Even tasks as simple as grasping a cup of coffee require a plethora of different processes that culminate in executing the corresponding action. These comprise, amongst others, perception of a stimulus in one’s environment, inferring its meaning, considering possible consequences, and making a decision about computing, executing, and monitoring the required action, all of which is accomplished remarkably rapidly and supposedly readily but, in fact, entails complex brain activity (see also Moravec, 1988, p. 15). One important ingredient of this scenario is the binding of stimulus and response features (S–R binding) enabling us to act in the world in goal-directed ways (Frings et al., 2020).

The theory of event coding (TEC; Hommel et al., 2001) assumes that stimulus and response features are *integrated* into so-called event files that interconnect stimulus (e.g., stimulus color or shape) and response features (e.g., the direction of a response or the effector; Hommel, 2004) into one combined representation (cf. Treisman & Gelade, 1980). Ample evidence shows that previously created event files are *retrieved* if at least one ingredient is repeated in a later episode (Hommel, 1998, 2004). How this retrieval affects performance depends on the relationship between the retrieved and the present combination of stimulus and response features: while performance (sometimes) improves in trials where all features of an event file repeat (benefits), performance is impaired if some but not all features are repeated (costs; Henson et al., 2014; Hommel, 1998). This is likely due to the feature codes contained in the event file conflicting with the features and feature combinations of the present episode (Frings & Moeller, 2012; Geissler et al., 2021; Hommel, 2004). This pattern of costs (and benefits) is referred to as *S–R binding effects.*^1^

Recent theorizing in the Binding and Retrieval in Action Control framework (BRAC; Frings et al., 2020) emphasizes that integration of features and the retrieval of features should be separated (cf., Hommel, 2022). In previous studies, it was often hard to disentangle whether experimental factors that successfully modify the size of such S–R binding effects were actually affecting feature integration proper, or the retrieval of integrated features, or both of these processes. There already exist several studies that demonstrate that disentangling both processes is possible and necessary (task instructions: Hommel et al., 2014; Memelink & Hommel, 2013; Mocke et al., 2020; temporal expectation: Schmalbrock & Frings, 2021; or perceptual salience: Schmalbrock et al., 2021). The present study aimed at disentangling these two processes with respect to S–R binding effects including task-irrelevant feature codes. There is good evidence that participants do not only integrate nominally task-relevant information about stimuli and responses into event files but also consider task-irrelevant information, like distractors in flanker-like setups (so-called distractor–response binding, DRB: Frings et al., 2007). This shows that irrelevant information is not only integrated but also retrieved. Here, we investigated whether the integration and retrieval processes involved in the S–R binding effects of irrelevant stimuli could be disentangled by manipulating stimulus predictability.

### Predictability and S–R binding

Research from adjacent fields demonstrates that *predictability* plays an important role in the processing of distractors. For example, the disrupting effect of salient distractors on search efficiency only emerges when salient trials are sufficiently rare (i.e., 20% of all trials). If salient distractors appear more often (i.e., 80% of all trials), participants can use their prediction of the next trial to prohibit attentional capture by salient distractors (Geyer et al., 2008). A study from the related flanker literature found that perfect predictability reduces the flanker effect substantially (Frings et al., 2019). Specifically, participants were presented with a target letter that could change randomly from trial to trial while irrelevant flanking letters were also present. Target and flanker letters were either presented in the same or different colors. For one group of participants, target and flanker color changed randomly, while for a second group flanker color was blocked. That is, for each trial of one block, participants saw the target and flanker either always in the same color or always in different colors. Intriguingly, the flanker compatibility effect was reduced in the blocked condition.

Findings from the visual search literature suggest that predictability might modulate the extent of attention a stimulus receives. Authors often operationalize predictability as *statistical regularities* in their paradigms (see Theeuwes & Failing, 2020, Chapter 4.3 for an overview). For example, Wang and Theeuwes (2018b) presented participants with a search display that could (irregularly) also contain a salient distractor item that usually leads to large interference effects (e.g., Theeuwes, 1992). However, if the distractor was repeatedly presented at the same location (above chance), interference effects markedly declined. The authors concluded that participants managed to reduce the processing priority of this specific location so that high-priority stimuli (i.e., a salient stimulus) in that location could interfere less. This finding and the conclusion are now supported by several publications (e.g., Failing et al., 2019; Failing & Theeuwes, 2020; Goschy et al., 2014; Wang & Theeuwes, 2018a, 2018b, 2018c). Interestingly, this bias is implemented *proactively* (Huang et al., 2021; van Moorselaar & Theeuwes, 2022; Wang et al., 2019). That is, processing of the predictable location is systematically reduced even before the actual search display is presented (or when a different display is presented).

This is in line with ecological approaches to behavior. Considerations about *curiosity* by Berlyne (1949, 1960) suggest that uncertainty (i.e., low predictability) induces a cognitive state of curiosity that drives participants to explore. Thus, unpredictable stimulus dimensions receive more attention while high predictability dimensions do not. Taken together, mechanistic and ecological approaches suggest that attending to stimuli crucially depends on the predictability of stimuli.

We hypothesized that introducing an element of predictability might alter the S–R binding effect because highly predictable stimuli might receive less attention and less attended stimuli have been demonstrated to show smaller S–R binding effects (Moeller & Frings, 2014; Singh et al., 2018). In fact, several findings from the S–R binding effect literature suggest that the strength of the S–R binding effect depends on attention (Hommel et al., 2014; Moeller & Frings, 2014; Singh et al., 2018; but see Hommel, 2005). In these studies, increased attention allocation towards a stimulus via a spatial cue led to increased S–R binding effects while reduced attention leads to reduced S–R binding effects. Given the findings from the visual attention literature, this would suggest that participants in S–R binding paradigms might proactively withdraw attentional resources from predictable distractors due to the bias they implemented based on their recent experience. This, in turn, would then lead to reduced S–R binding effects because crucial attentional resources are lacking for distractor processing.

### The present study

S–R binding effects are typically investigated in sequential priming paradigms. Participants execute two consecutive responses to given stimuli in two separate displays. A (first) prime display and a (second) probe display, make up an experimental prime-to-probe episode (i.e., a trial). In the DRB paradigm that we used in this study, participants respond to a central target letter flanked by two identical distractors in the prime and probe display. As mentioned already, probe performance depends on the relationship between prime and probe display configuration, which is systematically varied in DRB studies: Prime and probe can both require the same or different responses (response relation), and prime and probe distractor identity can be the same or different (distractor relation). This leads to four possible combinations: *complete repetitions* with response repetition (RR) + distractor repetition (DR) trials (RRDR), *partial repetitions* with response repetition + distractor change (DC), or response change (RC) + distractor repetition trials (RRDC and RCDR, respectively), and *complete changes* with response change + distractor change trials (RCDC). The standard finding is that performance in partial repetition trials is worse than in complete repetition or change trials—the S–R binding effect (Hommel, 1998). Note that since the retrieving stimulus is a distractor, we refer to this effect (also) as distractor–response binding effect (DRB; Frings et al., 2007)

We manipulated predictability by blocking the predictability of the distractors in prime and probe displays (Experiment 1) or only in the prime or probe display (Experiments 2a and 2b). For Experiment 1, participants worked through two blocks of distractor repetition trials (one for each possible distractor identity) and two blocks of distractor change trials (one for each possible distractor identity in the prime and the other in the probe). Block order was counterbalanced over all participants. This ensured that distractors were maximally predictable throughout a block. Target identity varied unpredictably from trial to trial and from prime to probe. In Experiments 2a, 2b, and 3, the identity of the distractors was blocked only in the prime (Experiment 2a) or the probe (Experiments 2b and 3), while the distractor in the not-blocked display varied unpredictably. The target varied unpredictably in prime and probe. In all experiments, half of all trials in a block were RC trials, the other RR trials—in the *high predictability* condition. We compared the performance of this high predictability group with a second *low predictability*^2^ group that worked through the regular DRB task (intermixed presentation of the different trial types).

We expected DRB effects to be lower in the high predictability group compared with the low predictability group. In Experiment 1, we applied the predictability manipulation simultaneously to prime and probe displays, so that we could investigate the general effect of distractor predictability on the DRB effect. In Experiments 2a, 2b, and 3, we applied the predictability manipulation exclusively to the prime (Experiment 2a) or the probe (Experiments 2b and 3). To foreshadow the results, distractor predictability diminished DRB in Experiment 1, and we could pinpoint this effect to the retrieval mechanism in Experiments 2b and 3.

## Experiment 1

### Method

#### Participants

Sixty-four students from Trier University (53 female; 55 right-handed) with a median age of 21 years (range: 18–38 years) participated. They received partial course credits for their 0.5 hours of service. This study followed the ethical standards defined by Trier University. The sample size was calculated according to previous studies investigating effects of DRB effect, which typically led to medium-sized effects (*d*_*z*_ = 0.50). Thus, we planned to run at least *N* = 27 participants, leading to a power of 1 − *β* = 0.80 (assuming an alpha = 0.05; G*Power 3.1.9.2; Faul et al., 2007), in each group to definitively observe the basic S–R effect without pinpointing the size of the assumed interaction. To account for possible dropout, we recruited five additional participants spread out over the high and low predictability groups.

#### Design

For this experiment two within-participant factors and one between-participant factor were varied: response relation (within: response repetition vs. response change), distractor relation (within: distractor repetition vs. distractor change), and predictability (between: high vs. low).

#### Apparatus and stimuli

**Fig. 1:**
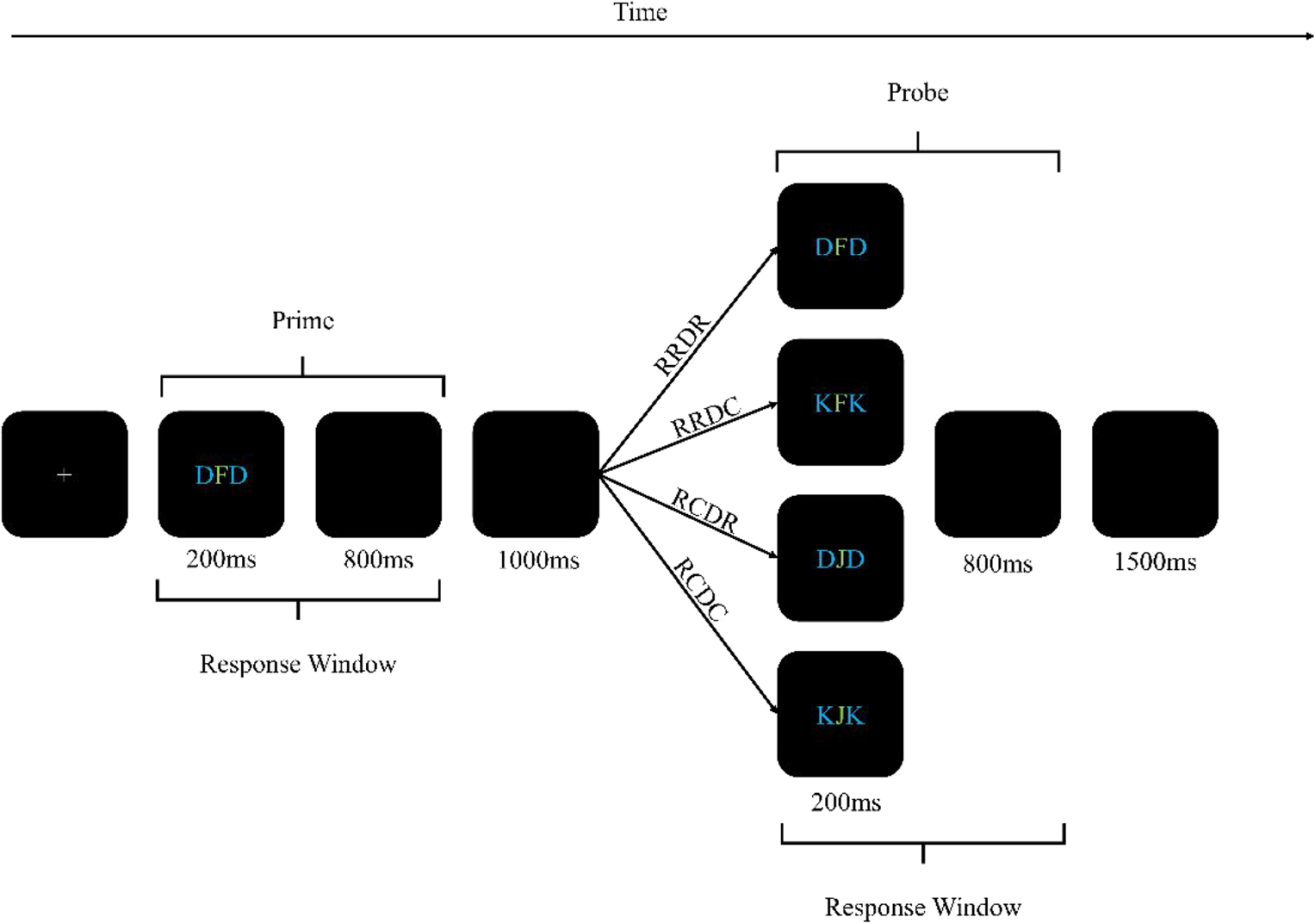
Procedure of the DRB task. Target letters were *F* or *J*; Distractor letters were *D* or *K*. Participants identified the central, green target letter. Probe display configuration was dependent on trial type and prime configuration. In response repetition trials (RR), the prime target was repeated as the probe target. In response change trials (RC), the probe target was different from the prime target. In distractor repetition trials (DR), the prime distractor was repeated as the probe distractor. In distractor change trials (DC), the probe distractor was different from the prime distractor. Note that in the low predictability conditions of Experiments 1, and 2a and 2b, trial type was determined randomly. In the high predictability condition, trial type was blocked (Experiment 1) or prime/probe distractor identity was blocked (Experiment 2a and 2b). Stimuli are not drawn to scale. (Color figure online)

The experiment was programmed using PsychoPy (Peirce et al., 2019; Version 03.01.2020) and run online via Pavlovia (Peirce & MacAskill, 2018). Two distinct displays were presented (see Fig. 1): a prime and probe display. In both displays, a string of three letters was presented at the screen center (Font: Arial; Font size 25 pixels). The central letter was presented in green (RGB_255_: 0, 128, 0) and was thus marked as the target. Target letters were either a *J* or an *F* (both presented equally often as prime and probe target). The two letters flanking the target letter were presented in blue (RGB_255_: 0, 0, 128) and thus marked as distractor letters. Distractor letters were either a *D* or a *K* (both presented equally often as prime and probe distractors; but see Procedure). All stimuli were presented on a black background (RGB_255_: 0, 0, 0).

#### Procedure

Participants registered for the study and received the study link from the recruitment platform used at Trier University (Sona Systems; sona-systems.com). Participants were instructed to place their left index finger on the *F* key and their right index finger on the *J* key. It was emphasized that responses were to be made as fast as possible while maintaining high accuracy. A training consisting of 20 trials was completed before the main experiment—here, participants received performance feedback after both prime and probe training displays (for a correct response: “Correct!”; for a wrong response: “Wrong!”; both in German language). After the training finished, participants only received feedback when they made an erroneous response after the display the error occurred in (“Wrong! Please respond as fast and correct as possible”; translated from German).

The task consisted of two consecutive responses. In both prime and probe displays, participants had to classify the identity of the centrally presented, green target letter. If the letter was an *F*, they responded with the *F* key; if the letter was a *J*, they responded with a *J* key press.

The experimental block consisted of 400 trials with a break after each 25-trials block (1× 400 trials in the low predictability group; 4× 100 trials in the high predictability group). A single trial consisted of the following chain of events: A trial began with a fixation mark (+) presented at the screen center for 1,000 ms. The fixation display was followed by the prime display. The prime display was presented for 200 ms with a response window of 1,000 ms, beginning with display onset. If a response was registered, the prime ended before 1,000 ms had elapsed. After the prime ended, a blank screen was presented for 1,000 ms. Then, the probe display was presented. The probe display was, again, presented for 200 ms with a response window of up to 1,000 ms. If a response was registered, the probe ended before 1,000 ms had elapsed. Each trial was separated from the next by a blank screen for 1,500 ms.

The two factors response relation and distractor relation were varied orthogonally. In response repetition trials, the same response required in the prime was also required in the probe. Vice versa, in response change trials a different response was required in prime and probe. In distractor repetition trials, the prime distractor was again presented in the probe. In distractor change trials, the prime distractor was different from the probe distractor.

The order in which the trials were presented differed between the high predictability and the low predictability condition. In the low predictability condition, all possible trial configurations derived from response relation, distractor relation, target, and distractor identity were presented randomly intermixed. In the high predictability condition, four separate blocks were presented, one for each possible trial configuration of distractor relation and distractor identity. Two blocks consisted of only distractor change trials and two blocks consisted only of distractor repetition trials. There were two blocks for each factor level of distractor relation and both possible distractor identities, leading to these four block types: Distractor repetition with only *D* as distractor; Distractor repetition with only *K* as distractor; Distractor change with *D* as a prime distractor and *K* as probe distractor; Distractor change with *K* as a prime distractor and *D* as probe distractor. Each block consisted of 50 response repetition trials and 50 response change trials. The order of blocks was further varied (see the Supplementary Material for an analysis of block order; in all three experiments block order did not affect any relevant effect).^3^ Two groups started with the two distractor repetition blocks and two groups started with the distractor change blocks. Each of the two groups started either with *D* as the first distractor letter or with *K* as the first distractor letter.

### Results

Data processing and analysis were done with R (R Core Team, 2019; R Version 3.6.1). The package dplyr (Wickham et al., 2019) was used for data processing and aggregation. The DRB effect was computed as the distractor repetition benefit in response repetition trials minus the distractor repetition cost in response change trials ([RRDC-RRDR]-[RCDC-RCDR]). Binding effects in the two different conditions were compared using a nonparametric Wilcoxon signed-rank test (Wilcoxon, 1992) supplemented by Bayesian *t* tests (Rouder et al., 2009), whose Bayes factors (*BF*_01_) quantify the evidence in favor of the null hypothesis relative to the evidence in favor of the alternative hypothesis. Values between 1 and 3 indicate weak/anecdotal evidence in favor of the null hypothesis, and values >3 indicate positive/strong evidence for the null hypothesis. Vice versa, values from 1 to .33 indicated weak/anecdotal evidence for the alternative hypothesis, and values <.33 indicate positive/strong evidence for the alternative hypothesis (Jarosz & Wiley, 2014). Bayes factors were computed using the package BayesFactor (Morey & Rouder, 2018).

#### Data processing

For the analysis of probe performance, only probe trials with correct answers in the prime were considered for error and reaction time (RT) analysis. Additionally, only trials with RTs longer than 200 ms and shorter than 1.5 interquartile ranges over the third quartile of each person’s RT distribution were analyzed (see Tukey, 1977). That is, 7% of all trials were excluded due to these constraints. For the analysis of probe RT, all trials with wrong probe responses were also excluded, that is an additional 6% of all trials (13% in total). See the Supplementary Material for the associated ANOVAs and Appendix 1 for a plot of mean RTs and error rates.

#### Reaction times

**Fig. 2:**
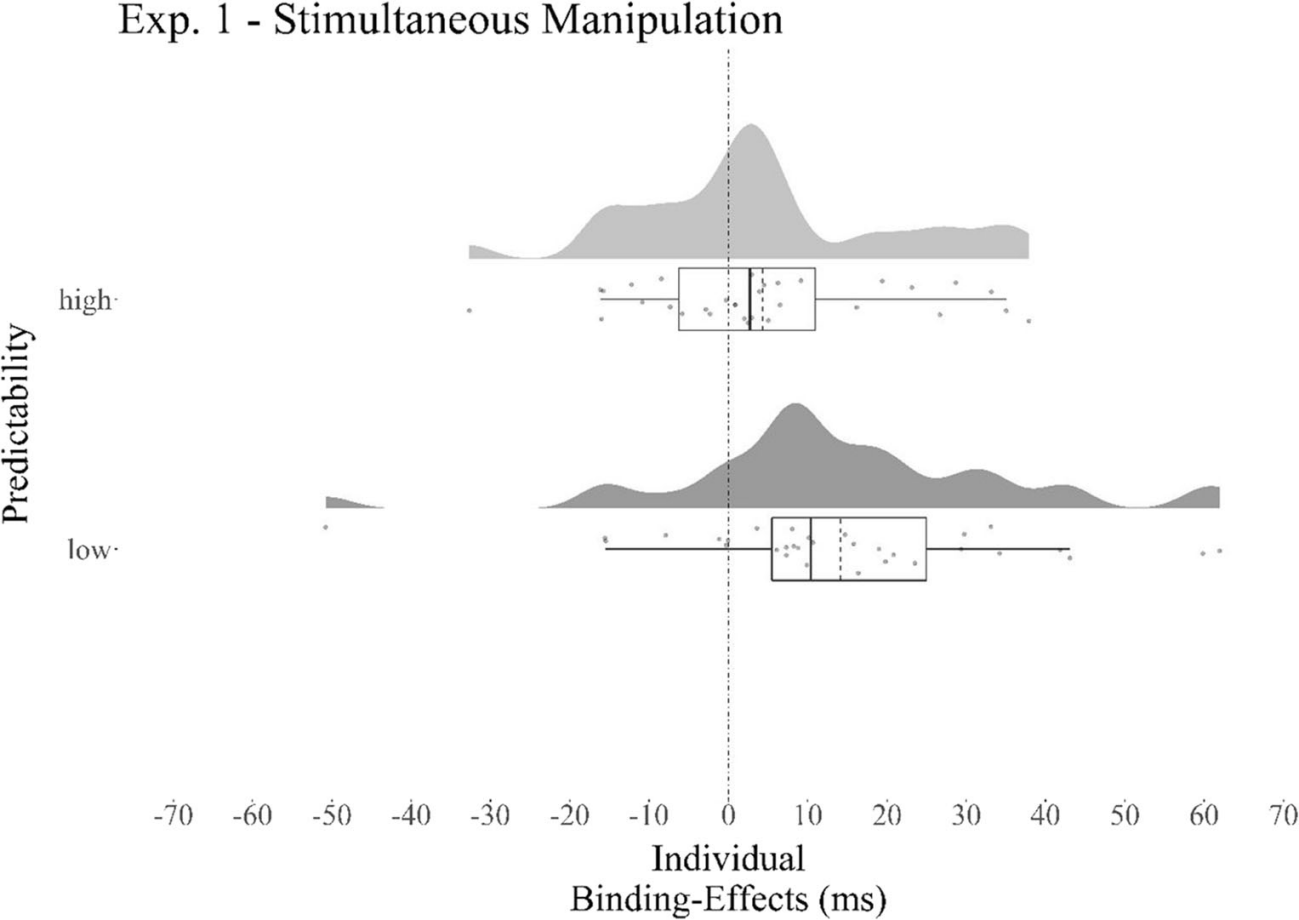
Individual binding effects for Experiment 1. Raincloud plots (Allen et al., 2019) for individual DRB effects in the high and low predictability condition for Experiment 1. The solid vertical line in each boxplot represents the median of the distribution; the dashed line represents the mean of the distribution. Upper and lower whiskers extend to the largest/smallest value above/below the respective hinge but at most 1.5 times the interquartile range above and below the third and first quartiles (McGill et al., 1978). Note that participants show lower binding effects when distractor stimuli are predictable

A Wilcoxon signed-rank test suggest that the DRB effects in the high predictability condition (*M* = 4 ms, *SD* = 16) were significantly lower than the DRB effects in the low predictability condition (*M* = 14 ms, *SD* = 22), *W* = 698, *p* = .006, *r* = .31, *BF*_01_ = 0.36 (one-tailed; see Fig. 2). A post hoc analysis evidenced that the DRB effects were significantly different from zero for the low predictability condition (one-tailed; *V* = 451, *p* < .001, *r* = .46, *BF*_01_ = 0.02) but not for the high predictability condition (one-tailed; *V* = 335, *p* = .095, *r* = .16, *BF*_01_ = 1.09).

#### Error rates

For the same analysis on probe error rates, only trials with correct prime responses but incorrect probe responses were considered (i.e., 6% of all trials were relevant for this analysis). A Wilcoxon signed-rank test suggest that the DRB effects in the high predictability condition (*M* = 2.52%, *SD* = 4.84) were not significantly lower than the DRB effects in the low predictability condition (*M* = 5.10%, *SD* = 8.56), *W* = 584, *p* = .170, *BF*_01_ = 0.85 (one-tailed). The post hoc analysis evidenced that the DRB effects were significantly different from zero for the low predictability condition (one-tailed, *V* = 6798, *p* < .001, *r* = .40 *BF*_01_ = 0.03), and for the high predictability condition (one-tailed, *V* = 6448, *p* < .001, *r* = .35 *BF*_01_ = 0.07).

### Discussion

In Experiment 1, we found a modulating effect of predictability on the DRB effect: DRB effects were stronger in the low predictability group compared with the high predictability group. Since the manipulation in Experiment 1 targeted both prime and probe (i.e., integration *and* retrieval), it is not possible to pinpoint whether our observation reflects an impact on integration, retrieval, or both. The BRAC framework emphasizes that a truly mechanistic understanding of the role of feature binding in action control requires the separation of effects on integration processes proper on the one hand and effects of the retrieval of event files on the other. To meet this requirement, we thus moved on to disentangle integration effects from retrieval effects of predictability. In Experiment 2a and 2b, we thus attempted to target integration (Experiment 2a) and retrieval (Experiment 2b) separately. We achieved this by presenting two blocks with fixed distractor identities in one display but unpredictable distractor identities in the other. In Experiment 2a, the prime distractor letters never changed throughout a block while the probe distractor letters changed unpredictably. In Experiment 2b, the probe distractor letters never changed throughout a block while the prime distractor letters changed unpredictably. We presented two blocks, one for each distractor identity used. Block order was counterbalanced over all participants.

Since we found a modulating influence of predictability on the DRB effect, we further expected to find a modulating influence of predictability on DRB effects in at least one of the two experiments. Specifically, DRB effects should be smaller in the high predictability condition compared with the low predictability condition.

## Experiment 2a and 2b

### Method

#### Participants

Sixty-four students participated in each experiment. No participant was excluded from Experiment 2a (47 female; 64 right-handed), their median age was 22 years (range: 19–37). Two participants were excluded from Experiment 2b because they did not comply with the instructions (they gave no response in any trial) bringing the final sample down to 62 participants (42 female; 57 right-handed); their median age was 22 years (range: 19–36). They received partial course credits for their 0.5 hours of service. This study followed the ethical standards defined by Trier University. The sample size for both experiments was calculated as in Experiment 1.

#### Design

For both experiments, two within-participant variables were varied: Response relation (repetition vs. change) and distractor relation (repetition vs. change). Additionally, one between-participant variable was varied: predictability of prime or probe distractors (high vs. low).

#### Apparatus and stimuli

Apparatus and stimuli were identical to those of Experiment 1.

#### Procedure

The procedure was identical to Experiment 1 except for the following changes: In the high predictability condition of Experiment 2a, two blocks were presented (one for each distractor identity, each 200 trials; in the low predictability condition, one block of 400 trials was presented). In each block, the same prime distractor letter was shown throughout the block while the probe distractor letter changed unpredictably. In the high predictability condition of Experiment 2b, two blocks were presented (one for each distractor identity). In each block, the same probe distractor letter was shown throughout the block while the prime distractor letter changed unpredictably. Half of the participants started with a block where the letter *D* was repeated, the other half started with the letter *K*. The second block was always the other, so far unused letter (see the Supplementary Material for an analysis of block order).^4^

### Results: Experiment 2a

#### Data processing

For the analysis of probe performance, only probe trials with correct answers in the prime were considered for error and RT analysis. Additionally, only trials with RTs longer than 200 ms and shorter than 1.5 interquartile ranges over the third quartile of each person’s RT distribution were analyzed (see Tukey, 1977). That is, 11% of all trials were excluded due to these constraints. For the analysis of probe RT, all trials with wrong probe responses were also excluded, that is an additional 7% of all trials (18% in total). See the Supplementary Material for the associated ANOVAs and Appendix 2 for a plot of mean RTs and error rates.

#### Probe reaction times

**Fig. 3:**
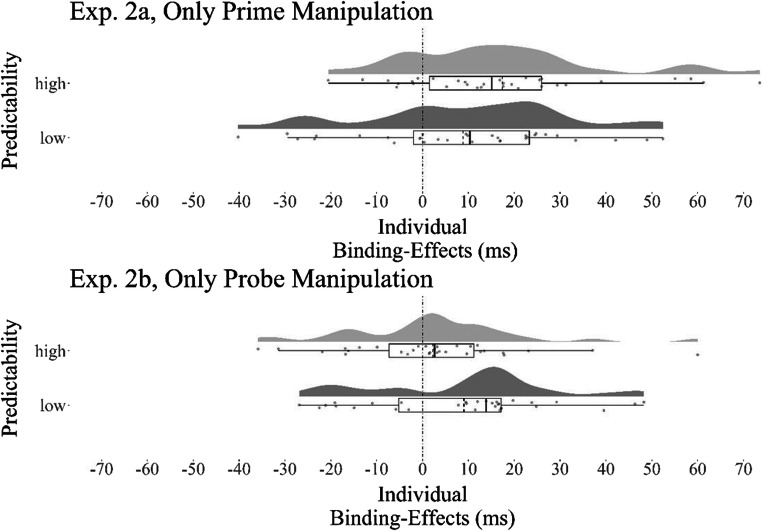
Individual binding effects for Experiment 2a and 2b. Raincloud plots (Allen et al., 2019) for individual binding effects in the high and low predictability condition for Experiment 2a (upper panel) and Experiment 2b (lower panel). The solid vertical line in each boxplot represents the median of the distribution; the dashed line represents the mean of the distribution. Upper and lower whiskers extend to the largest/smallest value above/below the respective hinge but at most 1.5 times the interquartile range above and below the third and first quartile (McGill et al., 1978). Note that in Experiment 2b, participants did not show DRB effects when distractors were highly predictable

A Wilcoxon signed-rank test suggested that the DRB effects in the high predictability condition (*M* = 17 ms, *SD* = 22) were not significantly lower than the DRB effects in the low predictability condition (*M* = 9 ms, *SD* = 23), *W* = 430, *p* = .138, *r* = .14, *BF*_01_ = 8.93 (one-tailed; see Fig. 3, upper panel). The post hoc analysis showed that the DRB effects were significantly different from zero for the low predictability condition (one-tailed, *V* = 372, *p* = .022, *r* = .25, *BF*_01_ = 0.32), and for the high predictability condition (one-tailed, *V* = 467, *p* < .001, *r* = .51, *BF*_01_ < 0.01).

#### Probe error rates

For the same analysis on probe error rates, only trials with correct prime responses but incorrect probe responses were considered (i.e., 7% of all trials were relevant for this analysis).

A Wilcoxon signed-rank test suggested that the DRB effects in the high predictability condition (*M* = 6%, *SD* = 8) were not significantly lower than the DRB effects in the low predictability condition (*M* = 3%, *SD* = 8), *W* = 446, *p* = .191, *BF*_01_ = 7.99 (one-tailed). The post hoc analysis evidenced that the DRB effects were significantly different from zero for the low predictability condition (one-tailed, *V* = 369, *p* = .007, *r* = .31, *BF*_01_ = 0.25), and for the high predictability condition (one-tailed, *V* = 451, *p* < .001, *r* = .46, *BF*_01_ = 0.01).

### Results: Experiment 2b

#### Data processing

For the analysis of probe performance, only probe trials with correct answers in the prime were considered for error and RT analysis. Additionally, only trials with RTs longer than 200 ms and shorter than 1.5 interquartile ranges over the third quartile of each person’s RT distribution were analyzed (see Tukey, 1977). That is, 12% of all trials were excluded due to these constraints. For the analysis of probe RT, all trials with wrong probe responses were also excluded, that is an additional 7% of all trials (19% in total). See the Supplementary Material for the associated ANOVAs and Appendix 2 for a plot of mean RTs and error rates.

#### Probe reaction times

A Wilcoxon signed-rank test suggested that the DRB effects in the high predictability condition (*M* = 2 ms, *SD* = 19) were significantly lower than the DRB effects in the low predictability condition (*M* = 9 ms, *SD* = 20), *W* = 600, *p* = .047, *r* = .21, *BF*_01_ = 0.96 (one-tailed; see Fig. 3, lower panel). Post hoc analysis evidenced that the DRB effects were significantly different from zero for the low predictability condition (one-tailed, *V* = 357, *p* = .032, *r* = .27, *BF*_01_ = 0.17), but not for the high predictability condition (one-tailed, *V* = 285, *p* = .480, *r* = .09, *BF*_01_ = 2.90).

#### Probe error rates

For the same analysis on probe error rates, only trials with correct prime responses but incorrect probe responses were considered (i.e., 7% of all trials were relevant for this analysis).

A Wilcoxon signed-rank test suggested that DRB effects in the high predictability condition (*M* = 3%, *SD* = 7) were not significantly lower than DRB effects in the low predictability condition (*M* = 5%, *SD* = 8), *W* = 539, *p* = .209, *r* = 0.10, *BF*_01_ = 1.63 (one-tailed). The post hoc analysis evidenced that the DRB effect were significantly different from zero for the low predictability condition (one-tailed, *V* = 422, *p* < .001, *r* = .45, *BF*_01_ = 0.05), and for the high predictability condition (one-tailed, *V* = 346, *p* = .028, *r* = .24, *BF*_01_ = 0.40).

### Discussion

In Experiment 2a and 2b, our goal was to further pinpoint the modulating effect predictability has on the DRB effect. That is, whether predictability affects integration, retrieval, or both. The fact that our predictability manipulation affected the size of the DRB effect only when applied to distractors in the probe trial, but not when applied to distractors in the prime trial, strongly suggests that the effect of distractor predictability does not reflect integration (Experiment 2a) but only retrieval (Experiment 2b).

Note that the comparison between high and low predictability conditions in Experiment 2b was only significant on the one-tailed level. On the one hand, using one-tailed testing can clearly be justified based on both the theoretical considerations motivating the present study and the empirical findings obtained in Experiment 1. On the other hand, however, one may ask why the obtained effect was not stronger. To some degree, one could have expected that restricting our manipulation to only one of the two displays would weaken our effects due to encoding specificity (Laub & Frings, 2020). That is, increasing dissimilarity between prime and probe displays should indeed reduce DRB effects and in fact any retrieval-based effect. Thus, by increasing encoding specificity we introduced more noise into the data, which is unfortunate but unavoidable for reaching our theoretical goals. To reduce the noise in our data at least somewhat, we aimed to replicate the findings of Experiment 2b in Experiment 3. Since the added noise of our manipulation worked on top of the already noisy online environment, we ran our replication in an offline setting.

## Experiment 3

### Method

#### Participants

Sixty-four students were recruited for this experiment. Two participants were lost due to a technical error, bringing the final sample down to 62 participants (47 female, 15 male; 56 right-handed, six left-handed). They received partial course credits for their 0.5 hours of service. This study followed the ethical standards defined by Trier University. The sample size was calculated as in Experiment 1.

#### Design

The experiment’s design was identical to that of Experiment 2b.

#### Apparatus and stimuli

Apparatus and Stimuli were identical to Experiment 2b with the important difference that Experiment 3 was run in an offline laboratory setting. That is, participants were individually tested in sound-proof chambers and were seated approximately 50 cm away from a 22-inch display monitor (60 Hz refresh rate, 1,680 × 1,050-pixel resolution). To ensure temporal stability of luminance and color, the monitor was warmed up 5 min before the experiment begin (Poth & Horstmann, 2017).

#### Procedure

The procedure was identical to that of Experiment 2b (see the Supplementary Material for an analysis of block order).

### Results

#### Data processing

For the analysis of probe performance, only probe trials with correct answers in the prime were considered for error and RT analysis. Additionally, only trials with RTs longer than 200 ms and shorter than 1.5 interquartile ranges over the third quartile of each person’s RT distribution were analyzed (see Tukey, 1977). That is, 4% of all trials were excluded due to these constraints. For the analysis of probe RT, all trials with wrong probe responses were also excluded, that is an additional 4% of all trials (8% in total). See the Supplementary Material for the associated ANOVAs and Appendix 3 for a plot of mean RTs and error rates. The analysis followed the preregistered analysis plan.

#### Probe reaction times

**Fig. 4:**
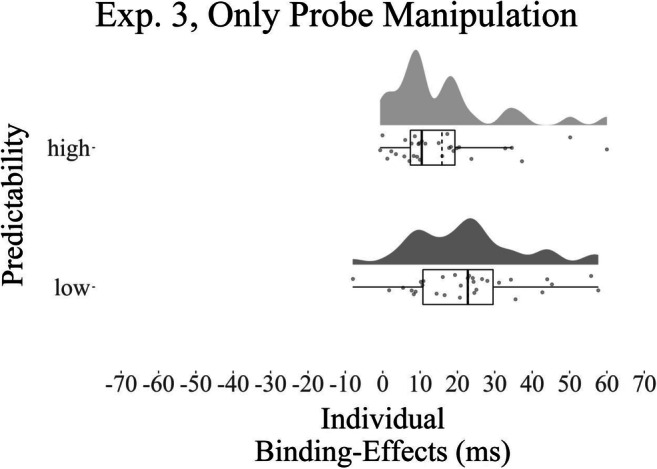
Individual binding effects for Experiment 3. Raincloud plots (Allen et al., 2019) for individual DRB effects in the high and low predictability condition for Experiment 3. The solid vertical line in each boxplot represents the median of the distribution; the dashed line represents the mean of the distribution. Upper and lower whiskers extend to the largest/smallest value above/below the respective hinge but at most 1.5 times the interquartile range above and below the third and first quartiles (McGill et al., 1978). Note that participants show lower S-R binding effects when distractor stimuli are predictable

A Wilcoxon signed-rank test suggested that the DRB effects in the high predictability condition (*M* = 16 ms, *SD* = 15) were significantly lower than the DRB effects in the low predictability condition (*M* = 23 ms, *SD* = 14), *W* = 341, *p* = .001, *r* = .30, *BF*_*01*_ = 0.91 (one-tailed; see Fig. 4). The post hoc analysis showed that the DRB effects were significantly different from zero for the low predictability condition (one-tailed, *V* = 493, *p* < .001, *r* = 1.05, *BF*_*01*_ < 0.01), and for the high predictability condition (one-tailed, *V* = 492, *p* < .001, *r* = 1.04, *BF*_*01*_ < 0.01).

#### Probe error rates

For the same analysis on probe error rates, only trials with correct prime responses but incorrect probe responses were considered (i.e., 4% of all trials were relevant for this analysis).

A Wilcoxon signed-rank test suggested that the DRB effects in the high predictability condition (*M* = 4%, *SD* = 5) were not significantly lower than the DRB effects in the low predictability condition (*M* = 5%, *SD* = 5), *W* = 535, *p* = .224, *r* = .10, *BF*_01_ = 2.74 (one-tailed). The post hoc analysis evidenced that the DRB effects were significantly different from zero for the low predictability condition (one-tailed, *V* = 479, *p* < .001, *r* = .81, *BF*_01_ < 0.01), and for the high predictability condition (one-tailed, *V* = 454, *p* < .001, *r* = .79, *BF*_01_ = 0.01).

## General discussion

The present study investigated the influence of predictability on DRB effects. Previous studies found that less attention is focused on predictable stimuli and that S–R binding effects hinge (at least partially) on attention. Against this background, we tested whether S–R binding effects might be reduced when stimulus predictability is high. We further tried to selectively attribute the effect of predictability to S–R feature integration and retrieval (Frings et al., 2020). We did so by first testing whether the DRB effect is sensitive to distractor predictability at all in Experiment 1. As this was the case, we selectively manipulated predictability with respect to the prime distractors at the probe distractors in Experiments 2a and 2b, respectively. We found that the impact of predictability was restricted to the predictability of probe distractors, suggesting that predictability manipulations affect the retrieval of event files but not their integration. In Experiment 3, we could replicate the finding of Experiment 2b in an offline setting—which further underlines the role of predictability for the retrieval process.

The findings of Experiment 2a confirm that integration seems to be a rather automatic process (Hommel, 2004), in the sense that it does not rely or depend on any particular top-down processes or attentional attitudes. Given the findings from the attention literature, higher predictability should decrease attention (e.g., Failing et al., 2019; Failing & Theeuwes, 2020; Goschy et al., 2014; Huang et al., 2021; van Moorselaar & Theeuwes, 2022; Wang et al., 2019; Wang & Theeuwes, 2018a, 2018b, 2018c), which should have reduced the impact of distractors. As this did not affect the S–R binding effect, it seems fair to assume that integration is not only automatic but also requires little attention to unfold (Hommel, 2005). The fact that we still observed DRB effects in the high predictability condition indicates that the distractors have been processed. Yet, since DRB effects did not differ between conditions, it is reasonable to assume that even superficial stimulus processing is sufficient for integration.

This does not seem to hold for the retrieval process, as predictable distractors apparently reduced the probability that the previous event file was retrieved (Experiments 2b and 3). Note also that the replication yielded less noisy data in terms of less overall errors and faster reaction times, which further underlines that replicating our findings offline lead to an overall cleaner estimate of the actual effects. As pointed out in the Discussion of Experiment 2b, we expected effects to be weaker in Experiment 2 compared with Experiment 1 due to the noise-increasing effect of encoding specificity. Nonetheless, given the reliable modulating effect of predictability in Experiment 1, we think it is safe to assume that even though encoding specificity might have lessened the strength of our modulating effect in Experiments 2b and 3, the sole source of the modulation must be the retrieval process. Given the ample evidence from the visual search literature, we suggest that predictability modulates event file retrieval via attention. This observation fits also well with other findings that retrieval is particularly sensitive to manipulations of attention (Ihrke et al., 2011; Schmalbrock et al., 2022; Schmalbrock & Frings, 2022). Several mechanisms might come into play here. For instance, as suggested in the Introduction, it has been shown that reduced stimulus variance dampens curiosity and thereby the processing of these stimuli (Frings et al., 2019). In addition, continuously ignoring the same distractor might lead to increased inhibition of such a stimulus and as a consequence this stimulus retrieves less (but see Giesen et al., 2012). Finally, perceptual habituation might play a role here, too (see Failing & Theeuwes, 2020; Narhi-Martinez et al., 2022, for an overview). It goes beyond the current paper to pinpoint which of these explanations affects event file retrieval here. At an overarching level, it seems clear that predictable distractors are processed in a way that they retrieve less.

Following findings from the attention literature, we assumed that predictability might play an important role in S–R binding—an assumption our results support. Further, we showed that both processes contributing to S–R binding effects are not equally affected by our predictability manipulation. Our findings are in line with recent theorizing (Frings et al., 2020) suggesting that experimentally disentangling the two processes that might contribute to S–R binding effects—integration and retrieval—is likely to deepen our understanding of the role of event files in action control. Our data suggest that integration of features is a rather automatic process that is not modulated by predictability while event file retrieval is. This fits with recent theorizing on the role of interindividual differences on S–R binding effects (Hommel, 2022).

### Supplementary information

ESM 1 (DOCX 55 kb)

## Figures and Tables

**Fig. 5:**
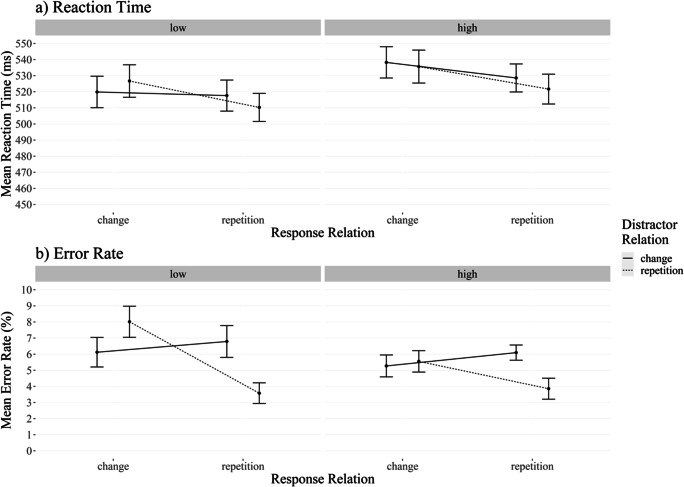
Mean reaction time and error rates for Experiment 1. Reaction time (**a**) and error rates (**b**) as a function of response relation (repetition vs. change), distractor relation (repetition vs. change), and predictability (high vs. low). Error bars indicate between-participants standard error of the mean

**Fig. 6:**
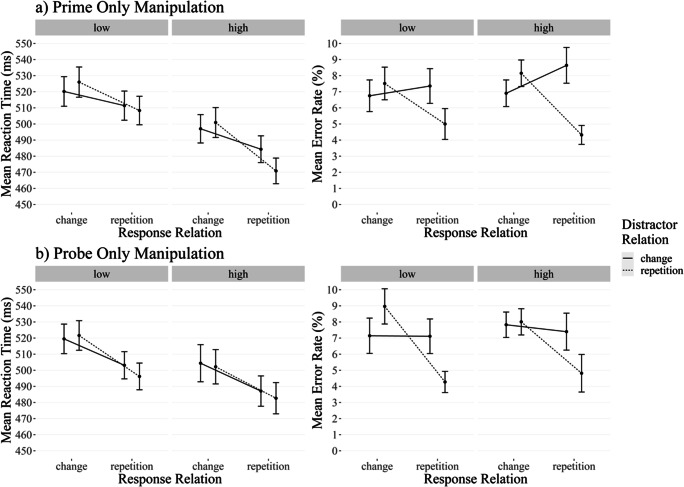
Mean reaction time and error rates for Experiments 2a and 2b. Reaction time and error rates as a function of response relation (repetition vs. change), distractor relation (repetition vs. change), and predictability (high vs. low) for Experiment 2a (**a**) and 2b (**b**). Error bars indicate between-participants standard error of the mean

**Fig. 7:**
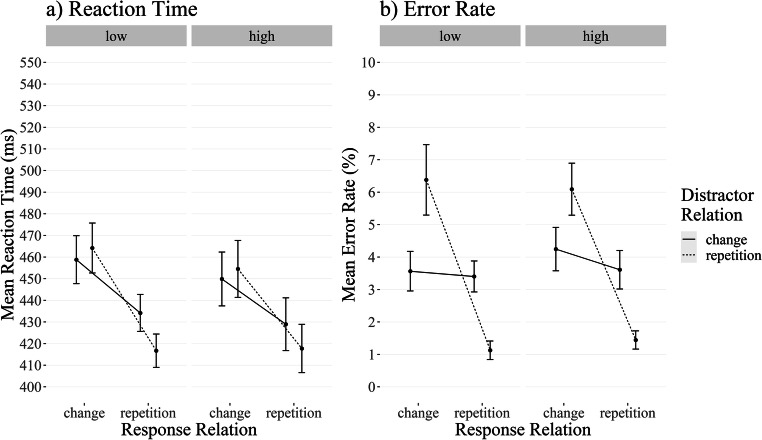
Mean reaction time and error rates for Experiment 3. Reaction time (**a**) and error rates (**b**) as a function of response relation (repetition vs. change), distractor relation (repetition vs. change), and predictability (high vs. low). Error bars indicate between-participants standard error of the mean

## Data Availability

The data for all experiments are available at PsychArchives (10.23668/psycharchives.8396). Experiment 3 was preregistered, and this preregistration can be found online (https://aspredicted.org/zd94a.pdf).

## Appendix 1

Fig. 5

## Appendix 2

Fig. 6

## Appendix 3

Fig. 7

## Footnotes

1 Note that we use the term *S–R binding effects* to reference the whole effect pattern that integration and retrieval contribute to. Other studies use the terms *binding* and *integration* interchangeably.

2 We chose this label instead of the “no predictability” label because although participants do not know which of two distractor letters will be shown, they at least knew that the upcoming distractor will be one of the two possible distractors.

3 *Block Order A*: (1) Distractor repetition D, (2) Distractor repetition K, (3) Distractor change D, (4) Distractor change K; *Block Order B*: (1) Distractor repetition K, (2) Distractor repetition D, (3) Distractor change K, (4) Distractor change D; *Block Order C*: (1) Distractor change K, (2) Distractor change D, (3) Distractor repetition D, (4) Distractor repetition K; *Block Order D*: (1) Distractor change D, (2) Distractor change K, (3) Distractor repetition K, (4) Distractor repetition D.

4 *Block Order A*: (1) D (2) K; *Block Oder B*: (1) K, (2) D.
