# Supplementary material for: Predictability reduces event file retrieval
Source: Atten Percept Psychophys. 2022 Dec 28;85(4):1073–87. doi: 10.3758/s13414-022-02637-6 (PMC10167154; doi:10.3758/s13414-022-02637-6)
Supplement: Supplementary file 1 — (DOCX 55 kb) [file 13414_2022_2637_MOESM1_ESM.docx]

**Supplementary Material**

Experimental conditions were compared using a repeated-measures analysis of variance (ANOVA) with type-III sums of square, using the ‘ezAnova’- function from the package ‘ez’ (Lawrence, 2016). We report two effect sizes for ANOVAs: ${\eta_{P}}^{2}$, and ${\eta_{G}}^{2}$ (Bakeman, 2005). The distractor-response binding effect is computed as the distractor repetition benefit in response repetition trials minus the distractor repetition interference in response change trials ([RRDC-RRDR]-[RCDC-RCDR]). Binding effects were compared using post-hoc t-tests complemented by Bayesian *t*-tests (Rouder et al., 2009) whose Bayes factor (${BF}_{01}$) quantify the evidence in favor of the null hypothesis relative to the evidence in favor of the alternative hypothesis. Bayes factors were computed using the package ‘BayesFactor’ (Morey & Rouder, 2018).

**Results ANOVA Experiment 1**

*Probe Reaction Time*

A 2 (response relation: repetition vs. change) x 2 (distractor relation: repetition vs. change) x 4 (block order: A vs. B vs. C. vs. D) mixed-effects ANOVA on probe RTs yielded no main effect for block order, *F*(3, 28) = 0.87, *p* = .470, ${\eta_{G}}^{2}$ = .08, ${\eta_{P}}^{2}$ = .08, indicating that there was no general performance difference between each group. No further interaction with block order reach significance, all *F*s < 1.3 and *p*s > .290. We, therefore, conclude that there was no influence of the order the trials in the high predictability condition were presented in and will exclude it henceforth from any following analysis.

A 2 (response relation: repetition vs. change) x 2 (distractor relation: repetition vs. change) x 2 (predictability: high vs. low) mixed-effects ANOVA on probe RTs yielded a significant interaction between response relation and distractor relation, *F*(1, 62) = 14.42, *p* < .001, ${\eta_{G}}^{2}$ < .01, ${\eta_{P}}^{2}$ = .19, indicating significant DRB effects. Intriguingly, this interaction was further modulated by predictability, resulting in a significant three-way interaction, *F*(1, 62) = 4.12, *p* = .047, ${\eta_{G}}^{2}$ < .01, ${\eta_{P}}^{2}$ = .06, suggesting that the DRB effect is modulated by the level of predictability (see **Figure 2**). This effect is further supplemented when Bayes factors are considered: A Welch two-sample *t*-test underlined that the DRB effects for the high predictability condition (*M* = 4 ms, *SD* = 16) were significantly lower from the DRB effect in the low predictability condition (*M* = 14 ms, *SD* = 22), two-tailed *t*(62) = 2.03, *p* = .047, *d_z_* = 0.25,${BF}_{01}$ = 0.70. Post-Hoc analysis evidenced that the DRB effects were significantly different from zero for the low predictability condition (two-tailed *t*(31) = 3.64, *p* = .001, *d_z_* = 0.64,${BF}_{01}$ = 0.03) but not for the high predictability condition (two-tailed *t*(31) = 1.47, *p* = .151, *d_z_* = 0.26,${BF}_{01}$ = 1.99).

Additionally, a main effect for response relation emerged, *F*(1, 62) = 19.37, *p* < .001, ${\eta_{G}}^{2}$ = .01, ${\eta_{P}}^{2}$ = .23. No further main effect or interaction reached significance, all *F*s < 1.16 and *p*s > .280.

*Probe Error Rate*

For the same analysis on probe error rates, only trials with correct prime responses but incorrect probe responses were considered (i.e., 6% of all trials were relevant error trials).

The same mixed-effects ANOVA including block order yielded no main effect for block order, *F*(3, 28) = 0.33, *p* = .805, ${\eta_{G}}^{2}$ = .02, ${\eta_{P}}^{2}$ = .03, indicating that there was no general performance difference between each group. No other main interaction including block order reached significance, we excluded the factor block order from the following analysis, all *F*s < 2.37 and *p*s > .09.

The mixed-effects ANOVA excluding block order, but including predictability yielded a significant interaction between response relation and distractor relation, *F*(1, 62) = 19.23, *p* < .001, ${\eta_{G}}^{2}$ =.05, ${\eta_{P}}^{2}$ = .24, again, indicating significant DRB effect. However, this interaction was not further modulated by predictability, *F*(1, 62) = 2.19, *p* = .144, ${\eta_{G}}^{2}$ = .01, ${\eta_{P}}^{2}$ = .03. A Welch two-sample *t*-test underlined that the DRB effects for the low predictability condition (*M* = 5.10 %, *SD* = 8.56) were not significantly different from the DRB effects in the high predictability condition (*M* = 2.52 %, *SD* = 4.84), two-tailed *t*(62) = 1.48, *p* = .145, *d_z_* = 0.19,${BF}_{01}$= 1.56. Post-Hoc analysis evidenced that the DRB effects were significantly different from zero for the low predictability condition (two-tailed *t*(31) = 3.37, *p* = .002, *d_z_* = 0.60,${BF}_{01}$ = 0.06) and also for the high predictability condition (two-tailed *t*(29) = 2.69, *p* = .006, *d_z_* = 0.52,${BF}_{01}$ = 0.15).

Additionally, several main effects emerged. A main effect for response relation was observed, *F*(1, 62) = 4.28, *p* = .043, ${\eta_{G}}^{2}$ = .02, ${\eta_{P}}^{2}$ = .06. Participants made more errors when responses changed from prime to probe (*M* = 6.24 %, *SD* = 4.70), compared to when the response repeated from prime to probe (*M* = 5.08 %, *SD* = 4.23). A main effect for distractor relation emerged, *F*(1, 62) = 4.57, *p* = .037, ${\eta_{G}}^{2}$ < .01, ${\eta_{P}}^{2}$ = .07. Participants made more errors when the distractor changed (*M* = 6.07 %, *SD* = 4.46), compared to when it repeated (*M* = 5.25 %, *SD* = 4.52). No further main effect or interaction reached significance, all *F*s < 2.20 and *p*s > .142.

**Results ANOVA Experiment 2a**

*Probe Reaction Times*

A 2 (response relation: repetition vs. change) x 2 (distractor relation: repetition vs. change) x 4 (block order: A vs. B) mixed-effects ANOVA on probe RTs yielded no main effect for block order, *F*(1, 30) = 0.11, *p* = .747, ${\eta_{G}}^{2}$ <.01, ${\eta_{P}}^{2}$ < .01, indicating that there was no general performance difference between each group. The interaction between block order and response relation reached significance, *F*(1, 30) = 6.60, *p* = .015, ${\eta_{G}}^{2}$ < .01, ${\eta_{P}}^{2}$ = .18. This is however of no concern to our research question. No further interaction with block order reach significance, all *F*s < 2.65 and *p*s > .11. We, therefore, conclude that there was no influence of the order the trials in the high predictability condition were presented in and will exclude it henceforth from any following analysis.

For the main analysis, a 2 (response relation: repetition vs. change) x 2 (distractor relation: repetition vs. change) x 2 (predictability: high vs. low) mixed-effects ANOVA on probe RTs yielded a significant interaction between response relation and distractor relation, *F*(1, 62) = 22.05, *p* < .001, ${\eta_{G}}^{2}$ <.01, ${\eta_{P}}^{2}$ = .26, indicating significant DRB effects.

Intriguingly, this interaction was not further modulated by predictability, *F*(1, 62) = 2.33, *p* = .132, ${\eta_{G}}^{2}$ < .01, ${\eta_{P}}^{2}$ = .03, suggesting that distractor-response integration is not modulated by the level of predictability (see **Figure 2**). A Welch two-sample *t*-test also underlined that the DRB effects for the high predictability condition (*M* = 17 ms, *SD* = 22) were not significantly lower compared to the DRB effects in the low predictability condition (*M* = 9ms, *SD* = 23), two-tailed *t*(62) = -1.53, *p* = .132, *d_z_* = 0.19,${BF}_{01}$ = 1.47. Post-Hoc analysis evidenced that the DRB effects were significantly different from zero for the low predictability condition (two-tailed *t*(31) = 2.21, *p* = .034, *d_z_* = 0.39,${BF}_{01}$ = 0.64) and also for the high predictability condition (two-tailed *t*(31) = 4.46, *p* < .001, *d_z_* = 0.79,${BF}_{01}$ < 0.01).

Additionally several main and interaction effects emerged. A main effect for response relation emerged, *F*(1, 62) = 42.04, *p* < .001, ${\eta_{G}}^{2}$ = .03, ${\eta_{P}}^{2}$ = .40. Participants responded faster when the response repeated (*M* = 494ms, *SD* = 51) compared to when it changed (*M* = 511ms, *SD* = 53). Further, a main effect for predictability emerged, *F*(1, 62) = 5.43, *p* = .023, ${\eta_{G}}^{2}$ = .07, ${\eta_{P}}^{2}$ = .08. Participants responded faster when the prime distractor predictability was high (*M* = 488ms, *SD* = 50) compared to when it was low (*M* = 516 ms, *SD* = 51). Also an interaction between response relation and predictability emerged, *F*(1, 62) = 5.47, *p* = .023, ${\eta_{G}}^{2}$ < .01, ${\eta_{P}}^{2}$ = .08. Responses were slowest when predictability was low and the response changed (*M* = 523 ms, *SD* = 52), somewhat faster when predictability was low and the response repeated (*M* = 510 ms, *SD* = 50), even faster when predictability was high and the response changed (*M* = 499 ms, *SD* = 51), and fastest when predictability was high and the response repeated (*M* = 478 ms, *SD* = 46). No further main effect or interaction reached significance, all *F*s < 2.34 and *p*s > .132.

*Probe Error Rates*

For the same analysis on probe error rates, only trials with correct prime responses but incorrect probe responses were considered (i.e., 7% of all trials were relevant error trials).

The same mixed-effects ANOVA including block order yielded no main effect for block order, *F*(1, 30) = 0.31, *p* = .581, ${\eta_{G}}^{2}$ <.01, ${\eta_{P}}^{2}$ = .01, indicating that there was no general performance difference between each group. No other main or interaction effect including block order reached significance, we excluded the factor block order from the following analysis, all *F*s < 0.94 and *p*s > .330.

The mixed-effects ANOVA excluding block order, but including predictability yielded a significant interaction between response relation and distractor relation, *F*(1, 62) = 19.92, *p* < .001, ${\eta_{G}}^{2}$ =.04, ${\eta_{P}}^{2}$ = .24, again, indicating significant DRB effects. However, this interaction was also not further modulated by predictability, *F*(1, 62) = 1.57, *p* = .214, ${\eta_{G}}^{2}$ <.01, ${\eta_{P}}^{2}$ = .02. A Welch two-sample *t*-test underlined that the DRB effects for the low predictability condition (*M* = 3%, *SD* = 8) were not significantly different from the DRB effects in the high predictability condition (*M* = 6%, *SD* = 8), two-tailed *t*(62) = -1.26, *p* = .214, *d_z_* = 0.16,${BF}_{01}$ = 2.01. Post-Hoc analysis evidenced that the DRB effects were significantly different from zero for the low predictability condition (two-tailed *t*(31) = 2.35, *p* = .025, *d_z_* = 0.42,${BF}_{01}$ = 0.49) and also for the high predictability condition (two-tailed *t*(31) = 3.91, *p* < .001, *d_z_* = 0.69,${BF}_{01}$= 0.02).

Additionally, a main effects for distractor relation was observed, *F*(1, 62) = 5.94, *p* = .018, ${\eta_{G}}^{2}$ =.01, ${\eta_{P}}^{2}$ = .09. Participants made more errors when the distractor changed from prime to probe (*M* = 7 %, *SD* = 6), compared to when the distractor repeated from prime to probe (*M* = 6 %, *SD* = 5). No other main effect or interaction reached significance, all *F*s < 2.33 and *p*s > .130.

**Results ANOVA Experiment 2b**

*Probe Reaction Times*

A 2 (response relation: repetition vs. change) x 2 (distractor relation: repetition vs. change) x 4 (block order: A vs. B) mixed-effects ANOVA on probe RTs yielded a main effect for block order, *F*(1, 29) = 5.14, *p* = .031, ${\eta_{G}}^{2}$ = .14, ${\eta_{P}}^{2}$ = .15. Participants in Group A (*M* = 472 ms, *SD* = 43) were significantly faster than Group B (*M* = 515 ms, *SD* = 62). Since this is a between-participant comparison and concerns a main effect, it is of no relevance to our main research question. The interaction between block order and response relation reached significance, *F*(1, 29) = 4.86, *p* = .036, ${\eta_{G}}^{2}$ < .01, ${\eta_{P}}^{2}$ = .14. This is however of no concern to our research question. No further interaction with block order reach significance, all *F*s < 0.07 and *p*s > .930. We, therefore, conclude that there was no influence of the order the trials in the high predictability condition were presented in and will exclude it henceforth from any following analysis.

For the main analysis, a 2 (response relation: repetition vs. change) x 2 (distractor relation: repetition vs. change) x 2 (predictability: high vs. low) mixed-effects ANOVA on probe RTs yielded a significant interaction between response relation and distractor relation, *F*(1, 60) = 5.29, *p* = .025, ${\eta_{G}}^{2}$ <.01, ${\eta_{P}}^{2}$ = .08, indicating significant S-R binding.

This interaction was not further modulated by predictability, *F*(1, 60) = 1.92, *p* = .171, ${\eta_{G}}^{2}$ < .01, ${\eta_{P}}^{2}$ = .03, suggesting that distractor-response retrieval is not modulated by the level of predictability (see **Figure 3**). A Welch two-sample *t*-test also underlined that the DRB effects for the high predictability condition (*M* = 2 ms, *SD* = 19) were not significantly lower compared to the DRB effects in the low predictability condition (*M* = 9 ms, *SD* = 20), two-tailed *t*(60) = 1.39, *p* = .171, *d_z_* = 0.18,${BF}_{01}$ = 1.73. Post-Hoc analysis evidenced that the DRB effects were significantly different from zero for the low predictability condition (two-tailed *t*(30) = 2.55, *p* = .016, *d* = 0.45,${BF}_{01}$ = 0.33), but not for the high predictability condition (two-tailed *t*(30) = 0.66, *p* = .661, *d* = 0.12,${BF}_{01}$ = 4.27).

Additionally several main and interaction effects emerged. A main effect for response relation emerged, *F*(1, 62) = 56.51, *p* < .001, ${\eta_{G}}^{2}$ = .04, ${\eta_{P}}^{2}$ = .49. Participants responded faster when the response repeated (*M* = 492 ms, *SD* = 50) compared to when it changed (*M* = 512ms, *SD* = 57). Further, a main effect for distractor relation emerged, *F*(1, 60) = 5.63, *p* = .021, ${\eta_{G}}^{2}$ < .01, ${\eta_{P}}^{2}$ = .09. Participants responded faster when the distractor repeated (*M* = 501 ms, *SD* = 54) compared to when it changed (*M* = 504 ms, *SD* = 55). No further main effect or interaction reached significance, all *F*s < 1.48 and *p*s > .220.

*Probe Error Rates*

For the same analysis on probe error rates, only trials with correct prime responses but incorrect probe responses were considered (i.e., 7% of all trials were relevant error trials).

The same mixed-effects ANOVA including block order yielded a main effect for block order, *F*(1, 29) = 6.86, *p* = .014, ${\eta_{G}}^{2}$ = .11, ${\eta_{P}}^{2}$ = .19. Participants in Group A (*M* = 9 %, *SD* = 6) made more errors than Group B (*M* = 5 %, *SD* = 4). Since this is a between-participant comparison and concerns a main effect, it is of no relevance to our main research question. No other interaction including block order reached significance, we excluded the factor block order from the following analysis, all *F*s < 2.90 and *p*s > .100.

The mixed-effects ANOVA excluding block order, but including predictability yielded a significant interaction between response relation and distractor relation, *F*(1, 60) = 13.90, *p* < .001, ${\eta_{G}}^{2}$ =.03, ${\eta_{P}}^{2}$ = .19, again, indicating significant DRB effects. However, this interaction was not further modulated by predictability, *F*(1, 60) = 0.91, *p* = .344, ${\eta_{G}}^{2}$ < .01, ${\eta_{P}}^{2}$ = .01. A Welch two-sample *t*-test underlined that the DRB effects for the low predictability condition (*M* = 5 %, *SD* = 8) were not significantly different from the DRB effect in the high predictability condition (*M* = 3 %, *SD* = 7), two-tailed *t*(60) = 0.95, *p* = .344, *d_z_* = 0.12,${BF}_{01}$ = 2.63. Post-Hoc analysis evidenced that the DRB effects were significantly different from zero for the low predictability condition (two-tailed *t*(30) = 3.13, *p* < .001, *d_z_* = 0.56,${BF}_{01}$ = 0.10) and also for the high predictability condition (two-tailed *t*(30) = 2.09, *p* = .045, *d_z_* = 0.38,${BF}_{01}$= 0.78).

Additionally, a main effects for distractor relation was observed, *F*(1, 60) = 4.17, *p* = .456, ${\eta_{G}}^{2}$ < .01, ${\eta_{P}}^{2}$ = .06. Participants made more errors when distractor changed from prime to probe (*M* = 7.37 %, *SD* = 6), compared to when the distractor repeated from prime to probe (*M* = 6.51 %, *SD* = 6). Also a main effects for response relation was observed, *F*(1, 60) = 9.71, *p* = .003, ${\eta_{G}}^{2}$ =.03, ${\eta_{P}}^{2}$ = .13. Participants made more errors when response changed from prime to probe (*M* = 8 %, *SD* = 5), compared to when the response repeated from prime to probe (*M* = 6 %, *SD* = 6). No other main effect or interaction reached significance, all *F*s < 0.70 and *p*s > .400.

**Results ANOVA Experiment 3**

*Probe Reaction Times*

A 2 (response relation: repetition vs. change) x 2 (distractor relation: repetition vs. change) x 4 (block order: A vs. B) mixed-effects ANOVA on probe RTs yielded no main effect for block order, *F*(1, 29) = 0.63, *p* = .435, ${\eta_{G}}^{2}$ =.02, ${\eta_{P}}^{2}$ = .02, indicating that there was no general performance difference between each group. No further interaction with block order reach significance, all *F*s < 0.17 and *p*s > .680. We, therefore, conclude that there was no influence of the order the trials in the high predictability condition were presented in and will exclude it henceforth from any following analysis.

For the main analysis, a 2 (response relation: repetition vs. change) x 2 (distractor relation: repetition vs. change) x 2 (predictability: high vs. low) mixed-effects ANOVA on probe RTs yielded a significant interaction between response relation and distractor relation, *F*(1, 60) = 106.43, *p* < .001, ${\eta_{G}}^{2}$ <.01, ${\eta_{P}}^{2}$ = .64, indicating significant DRB effects.

Intriguingly, this interaction was not further modulated by predictability, *F*(1, 60) = 3.47, *p* = .068, ${\eta_{G}}^{2}$ < .01, ${\eta_{P}}^{2}$ = .05, suggesting that S-R integration is not modulated by the level of predictability (see **Figure 2**). A Welch two-sample *t*-test also underlined that the DRB effects for the high predictability condition (*M* = 16 ms, *SD* = 15) were not significantly lower compared to the DRB effects in the low predictability condition (*M* = 23 ms, *SD* = 14), two-tailed *t*(59.69) = 1.86, *p* = .068, *d_z_* = 0.24, *BF_01_* = 0.91. Post-Hoc analysis evidenced that the DRB effects were significantly different from zero for the low predictability condition (two-tailed *t*(30) = 8.32, *p* < .001, *d_z_* = 1.49, *BF_01_* < 0.01) and also for the high predictability condition (two-tailed *t*(30) = 6.21, *p* < .001, *d_z_* = 1.11, *BF_01_* < 0.01).

Additionally, several main effects emerged. A main effect for response relation emerged, *F*(1, 60) = 84.21, *p* < .001, ${\eta_{G}}^{2}$ = .07, ${\eta_{P}}^{2}$ = .58. Participants responded faster when the response repeated (*M* = 424 ms, *SD* = 56) compared to when it changed (*M* = 457 ms, *SD* = 67). Further, a main effect for the distractor relation emerged, *F*(1, 60) = 33.99, *p* < .001, ${\eta_{G}}^{2}$ < .01, ${\eta_{P}}^{2}$ = .36. Participants responded faster when the prime distractor repeated (*M* = 438 ms, *SD* = 65) compared to when it changed (*M* = 443 ms, *SD* = 63). No further main effect or interaction reached significance, all *F*s < 2.94 and *p*s > .09.

*Probe Error Rates*

For the same analysis on probe error rates, only trials with correct prime responses but incorrect probe responses were considered (i.e., 4% of all trials were relevant error trials).

The same mixed-effects ANOVA including block order yielded no main effect for block order, *F*(1, 29) = 1.72, *p* = .199, ${\eta_{G}}^{2}$ = .03, ${\eta_{P}}^{2}$ = .03, indicating that there was no general performance difference between each group. No other main or interaction effect including block order reached significance, we excluded the factor block order from the following analysis, all *F*s < 1.40 and *p*s > .240.

The mixed-effects ANOVA excluding block order, but including predictability yielded a significant interaction between response relation and distractor relation, *F*(1, 60) = 57.64, *p* < .001, ${\eta_{G}}^{2}$ =.09, ${\eta_{P}}^{2}$ = .49, again, indicating significant DRB effects. However, this interaction was also not further modulated by predictability, *F*(1, 60) = 0.81, *p* = .371, ${\eta_{G}}^{2}$ <.01, ${\eta_{P}}^{2}$ = .01. A Welch two-sample *t*-test underlined that the DRB effects for the low predictability condition (*M* = 5%, *SD* = 5) were not significantly different from the DRB effects in the high predictability condition (*M* = 4%, *SD* = 4), two-tailed *t*(59.42) = 0.90, *p* = .371, *d_z_* = 0.11,${BF}_{01}$ = 2.74. Post-Hoc analysis evidenced that the DRB effects were significantly different from zero for the low predictability condition (two-tailed *t*(30) = 5.73, *p* < .001, *d_z_* = 1.03,${BF}_{01}$ < 0.01) and also for the high predictability condition (two-tailed *t*(30) = 4.98, *p* < .001, *d_z_* = 0.89,${BF}_{01}$< 0.01).

Additionally, a main effects for response relation was observed, *F*(1, 60) = 31.43, *p* < .001, ${\eta_{G}}^{2}$ =.12, ${\eta_{P}}^{2}$ = .34. Participants made more errors when the response repeated from prime to probe (*M* = 3 %, *SD* = 2), compared to when the response changed from prime to probe (*M* = 5 %, *SD* = 5). No other main effect or interaction reached significance, all *F*s < 0.49 and *p*s > .490.
